# Development and validation of the fracture risk scale home care (FRS-HC) that predicts one-year incident fracture: an electronic record-linked longitudinal cohort study

**DOI:** 10.1186/s12891-020-03529-2

**Published:** 2020-07-28

**Authors:** Caitlin McArthur, George Ioannidis, Micaela Jantzi, Jonathon D. Adachi, Lora Giangregorio, John Hirdes, Alexandra Papaioannou

**Affiliations:** 1 McMaster University, 1200 Main Street, Hamilton, Ontario L8S 4L8 Canada; 2 GERAS Centre for Aging Research, 88 Maplewood Avenue, 88 Maplewood Avenue, Hamilton, Ontario L8M 1W9 Canada; 3 University of Waterloo, 200 University Avenue West, Waterloo, Ontario N2L 3G1 Canada; 4 Schlegel-UW Research Institute for Aging Research, 250 Laurelwood Drive, Waterloo, Ontario N2J OE2 Canada

**Keywords:** Fractures, Home care, osteoporosis, older adults, interRAI, Cohort

## Abstract

**Background:**

Fractures have dire consequences including pain, immobility, and death. People receiving home care are at higher risk for fractures than the general population. Yet, current fracture risk assessment tools require additional testing and assume a 10-year survival rate, when many die within one year. Our objectives were to develop and validate a scale that predicts one-year incident hip fracture using the home care resident assessment instrument (RAI-HC).

**Methods:**

This is a retrospective cohort study of linked population data. People receiving home care in Ontario, Canada between April 1st, 2011 and March 31st, 2015 were included. Clinical data were obtained from the RAI-HC which was linked to the Discharge Abstract Database and National Ambulatory Care Reporting System to capture one-year incident hip fractures. Seventy-five percent (*n* = 238,011) of the sample were randomly assigned to a derivation and 25% (*n* = 79,610) to a validation sample. A decision tree was created with the derivation sample using known fracture risk factors. The final nodes of the decision tree were collapsed into 8 risk levels and logistic regression was performed to determine odds of having a fracture for each level. c-Statistics were calculated to compare the discriminative properties of the full, derivation, and validation samples.

**Results:**

Approximately 60% of the sample were women and 53% were 80 years and older. A total of 11,526 (3.6%) fractures were captured over the 1-year time period. Of these, 5057 (43.9%) were hip fractures. The proportion who experienced a hip fracture in the next year ranged from 0.3% in the lowest risk level to 5.2% in the highest risk level. People in the highest risk level had 18.8 times higher odds (95% confidence interval, 14.6 to 24.3) of experiencing a hip fracture within one year than those in the lowest. c-Statistics were similar for the full (0.658), derivation (0.662), and validation (0.645) samples.

**Conclusions:**

The FRS-HC predicts hip fracture over one year and should be used to guide clinical care planning for home care recipients at high risk for fracture. Our next steps are to develop a fracture risk clinical assessment protocol to link treatment recommendations with identified fracture risk.

## Background

Worldwide, there has been a shift from institutional models of care (i.e., long-term care) to supporting older adults in their own homes. The consequence of the shift is that an increasing number of frail, medically-complex older adults are reliant on support from service such as home care which is defined as receiving nursing or professional services (e.g., physical or occupational therapy) for 60 days or more within a person’s home [1]. Because they are more medically complex, people receiving home care experience a higher incidence of negative events such as falls and fractures than the average population [2], further increasing their risk of functional dependence, institutionalization and mortality. The incidence of hip fracture for people receiving home care is high, at 24.4 per 1000 person-years [3] compared to 5.7 per 1000 person-years in the average population [4]. Fractures pose a significant burden to the health, quality of life, and mortality of older adults receiving home care services [3]. After a hip fracture, 25% of people require institutionalization [5], and over 20% will die [6]. Hip fractures also carry a significant economic burden; the costs associated with fractures in home care in Canada are $274 million [7]. Home care clients at risk for fractures must be identified and strategies must be implemented to prevent the loss of mobility and independence, and increased risk of death.

Fracture risk is commonly identified through risk assessment tools, such as the FRAX [8]. However, current fracture assessment tools may not be valid or generalizable for some medically complex home care recipients. First, current risk assessment tools do not capture potential risk factors that may be more relevant for assessing risk among the home care population (e.g., cognitive impairment, multi-morbidity and falls risk). Fracture risk outputs may not provide accurate estimates for home care recipients with multiple comorbidities. Current assessment tools rely on data often unavailable in routine home care assessments, such as bone mineral density, adding to the assessment burden of this sector. Finally, imminent fracture risk (i.e., within the next year) must be the target for fracture risk assessment with the vulnerable home care population. As many as 17% of home care clients die within one year from admission [2]. Risk estimates with a longer prediction timeframe will underestimate imminent fracture risk and lack the urgency for prevention strategies to be implemented.

The Resident Assessment Instrument – Home Care (RAI-HC) [9] is a comprehensive, standardized tool implemented as part of routine clinical practice across several Canadian provinces. The RAI-HC is completed upon admission into a long-stay home care programs and includes person-level data elements and outcome scores [9]. Outputs from the RAI-HC can be used to guide practice and identify home care recipients at risk for negative events or outcomes, such as fractures. The RAI-HC is routinely collected for all long-stay home care recipients in Canada and internationally meaning that fracture risk identification could be automatically incorporated into daily practice without additional documentation burden. Our objectives were to develop and validate a scale that predicts one-year incident hip fracture using the home care resident assessment instrument (RAI-HC). Our team has previously developed a Fracture Risk Scale for long-term care using a similar assessment system (MDS 2.0) [10]. However, given the observed difference in fracture incidence [11] and population characteristics [12] between home and long-term care, we considered it important to develop a tool specific to the unique attributes of home care recipients. Further, there continues to be large care gap for people identified at high fracture risk [13] despite bone mineral density often being available in primary care.

## Methods

### Study design

**Fig. 1:**
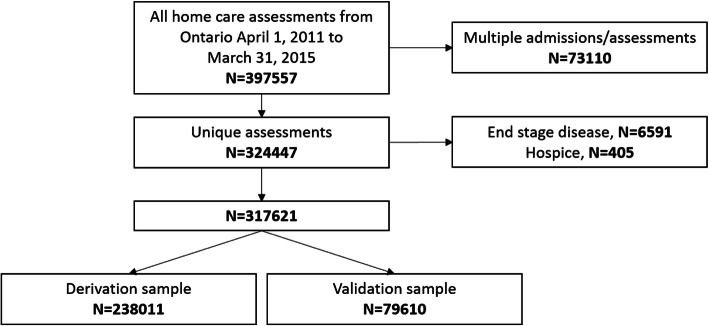
Study sample flow diagram

This is a retrospective cohort study of linked population data. People receiving home care in Ontario, Canada between April 1st, 2011 and March 31st, 2015 were included; home care recipients identified as end of life or receiving hospice care were excluded. The final sample size included 317,621 home care recipients. Seventy-five percent (*n* = 238,011) of the home care recipients were randomly assigned to a derivation and 25% (*n* = 79,610) to a validation sample (Fig. 1). Home care recipients were classified as experiencing or not experiencing an incident hip fracture over the 1-year follow-up period. We chose hip fracture as our target variable as they are the most common type of fracture that come to clinical attention [11], the negative sequelae of hip fractures often outweigh those of other types of fractures, other prediction models often use hip fractures as the target outcome, and to be consistent with our previously developed Fracture Risk Scale for long-term care [10].

### Data sources

Clinical data were obtained from the RAI-HC. The RAI-HC is a valid and reliable [14] standardized assessment that is completed upon admission for all recipients into long-stay home care programs in Ontario, Canada. The RAI-HC is completed by trained assessors who gather information from recipients, their family members and health care providers, and through chart review. It includes individual data elements and outcomes scores on over 200 health and social characteristics. RAI-HC data were linked to the Discharge Abstract Database (DAD) and National Ambulatory Care Reporting System (NACRS) to capture incident hip fracture. The DAD captures inpatient hospital stay data while the NACRS captures emergency department visits [15, 16]. Incident fracture was captured based on the definition of the Public Health Agency of Canada in the Revised Framework for National Surveillance on Osteoporosis and Osteoporosis-Related Fractures [17]. Using International Classification of Disease 10 codes, home care recipients were coded as having a fractures within 1-year of their admission assessment [hip (S72.0, S72.1 S72.2), spine (S22.0, S22.1 S 2.0, S32.8), humerus (S42.2), forearm (S52.x, S62.x), and pelvis (S32.1, S32.2, S32.4, S32.5, S32.8)]. Our target outcome was the first fracture diagnosis within one year and we did not include multiple admissions or discharges for the same diagnoses.

### Statistical analyses

Clinical characteristics were expressed in count and per cent for categorical variables, and we modelled the univariate associations (odds ratio and 95% confidence interval) between the final included risk factors and hip fracture incidence via multiple logistic regression models. We created a decision tree with the 75% derivation sample to predict incident hip fracture within one year of admission to home care using known fracture risk factors identified by previous literature [3, 10] and expert opinion. Decision trees are advantageous over standard additive models of risk because they allow for detection of complex interactions, identification of small but important groups at higher risk for the outcome, and inclusion of non-linear relationships [18, 19]. One year was chosen as the target time frame given the high one-year mortality rate in home care [2] and to be consistent with our previously developed Fracture Risk Scale for long-term care [10]. Items and scales were retained based on significant association with incident hip fractures, relevance of the items, and face validity as determined by a clinical expert panel. In SAS Enterprise Miner V.13.1 (SAS Institute), χ^2^ Automatic Interaction Detection used recursive partitioning to create the decision tree [20]. The final decision tree was validated at a meeting with clinical experts. Leaves of the final decision tree were combined to create the final risk levels of the scale based on examination by the expert panel of 1) the risk profiles; 2) incidence of hip fracture; and 3) proportion of the population in each leaf and risk level. An interactive decision tree analysis approach including clinical expert insight guided by statistical evidence was chosen to ensure the final tool had clinical relevance and would be applicable.

The final nodes of the decision tree were collapsed into eight risk levels and logistic regression was performed to determine the odds of having a fracture within one year of admission to home care for each level. c-Statistics were calculated to compare the discriminative properties of the full, derivation, and validation samples. All statistical analyses were completed in in SAS V9.3 (SAS Institute).

## Results

The population characteristics of the derivation, validation, and combined dataset are in Table 1. In the combined sample, 60% of the population were female and 53% were over the age of 80 years. There were 11,526 (3.6%) incident fractures within one year from admission to home care, of which 5057 (43.9%) were hip and 6959 (56.1%) were other locations (wrist, spine, humerus, pelvis). Population characteristics and fracture incidence were similar across the derivation, validation, and full sample datasets. Univariate odds ratios for the final included risk factors are found in Table 2.

**Table 1:** Characteristics of the full, derivation, and validation samples

Characteristic:	Full sample *N* = 317,621	Derivation sample *N* = 238,011	Validation sample *N* = 79,610
	N (%)	N (%)	N (%)
New fractures within one year from initial assessment	11,526 (3.6)	8679 (3.7)	2847 (3.6)
Hip	5057 (1.6)	3822 (1.6)	1235 (1.6)
Other fracture (spine, pelvis, humerus, wrist)	6959 (2.2)	5228 (2.2)	1731 (2.2)
Age group			
18 to 50	14,354 (4.5)	10,693 (4.5)	3661 (4.6)
50 to 64	37,652 (11.9)	28,297 (11.9)	9355 (11.8)
64 to 80	96,429 (30.4)	72,222 (30.4)	23,207 (30.4)
80+	169,137 (53.3)	126,763 (52.3)	42,374 (53.2)
Female	191,510 (60.3)	14,365 (60.3)	48,045 (60.4)
Unsteady gait	216,283 (68.1)	162,021 (68.1)	54,262 (68.2)
Wandering	10,165 (3.2)	7695 (3.2)	2470 (3.1)
Tobacco use	27,793 (8.8)	20,772 (8.7)	7021 (8.8)
Fall in last 180 days	143,666 (45.2)	107,588 (45.2)	36,074 (45.3)
Previous fracture in the last 180 days	43,569 (13.7)	32,564 (13.7)	11,005 (13.8)
Transfer ability			
Independent (0)	207,607 (65.4)	155,698 (65.4)	51,909 (65.2)
Supervision or set up help (1, 2)	43,085 (13.6)	32,292 (13.6)	10,793 (13.6)
Limited assistance (3)	28,550 (9.0)	21,244 (8.9)	7306 (9.2)
Extensive or maximal assistance (4, 5)	27,608 (8.7)	20,750 (8.7)	6858 (8.6)
Total dependence or did not occur (6, 8)	10,771 (3.4)	8027 (3.4)	2744 (3.4)
Primary mode of locomotion indoors			
No aid	117,271 (36.9)	88,060 (37.0)	29,209 (36.7)
Cane or walker	158,444 (49.9)	118,524 (49.8)	39,918 (50.1)
Scooter, wheelchair, or did not occur	41,911 (13.2)	31,427 (13.2)	10,483 (13.2)
Cognitive performance scale			
Intact (0)	107,057 (33.7)	80,222 (33.7)	26,833 (33.7)
Mild impairment (1, 2)	162,604 (51.2)	121,648 (51.1)	40,954 (51.4)
Moderate impairment (3, 4)	34,339 (10.8)	25,883 (10.9)	8455 (10.6)
Severe impairment (5, 6)	13,626 (4.3)	10,258 (4.3)	3368 (4.2)

**Table 2:** Univariate associations between risk factors and one-year incident fractures for the full sample

Characteristic:	Odds ratio (95% Confidence interval)
Age 70+ (REF = < 70)	3.5 (3.2 to 3.9)
Female (REF = female)	1.5 (1.4 to 1.6)
Unsteady gait (REF = steady gait)	1.6 (1.5 to 1.7)
Uses gait aid (REF = does not use gait aid)	1.5 (1.5 to 1.6)
Wandering (REF = no wandering)	2.5 (2.2 to 2.8)
Tobacco use (REF = no tobacco use)	1.0 (0.9 to 1.1)
Fall in last 180 days (REF = no fall)	1.6 (1.5 to 1.7)
Previous fracture in the last 180 days (REF = no fracture)	1.5 (1.4 to 1.6)
Dependent in transfers (REF = independent)	0.5 (0.4 to 0.6)
Ambulatory indoors with or without gait aid (REF = non-ambulatory)	1.3 (1.2 to 1.4)
Has cognitive impairment (CPS > 0) (REF = no cognitive impairment)	2.2 (2.0 to 2.3)

### Decision tree model

**Fig. 2:**
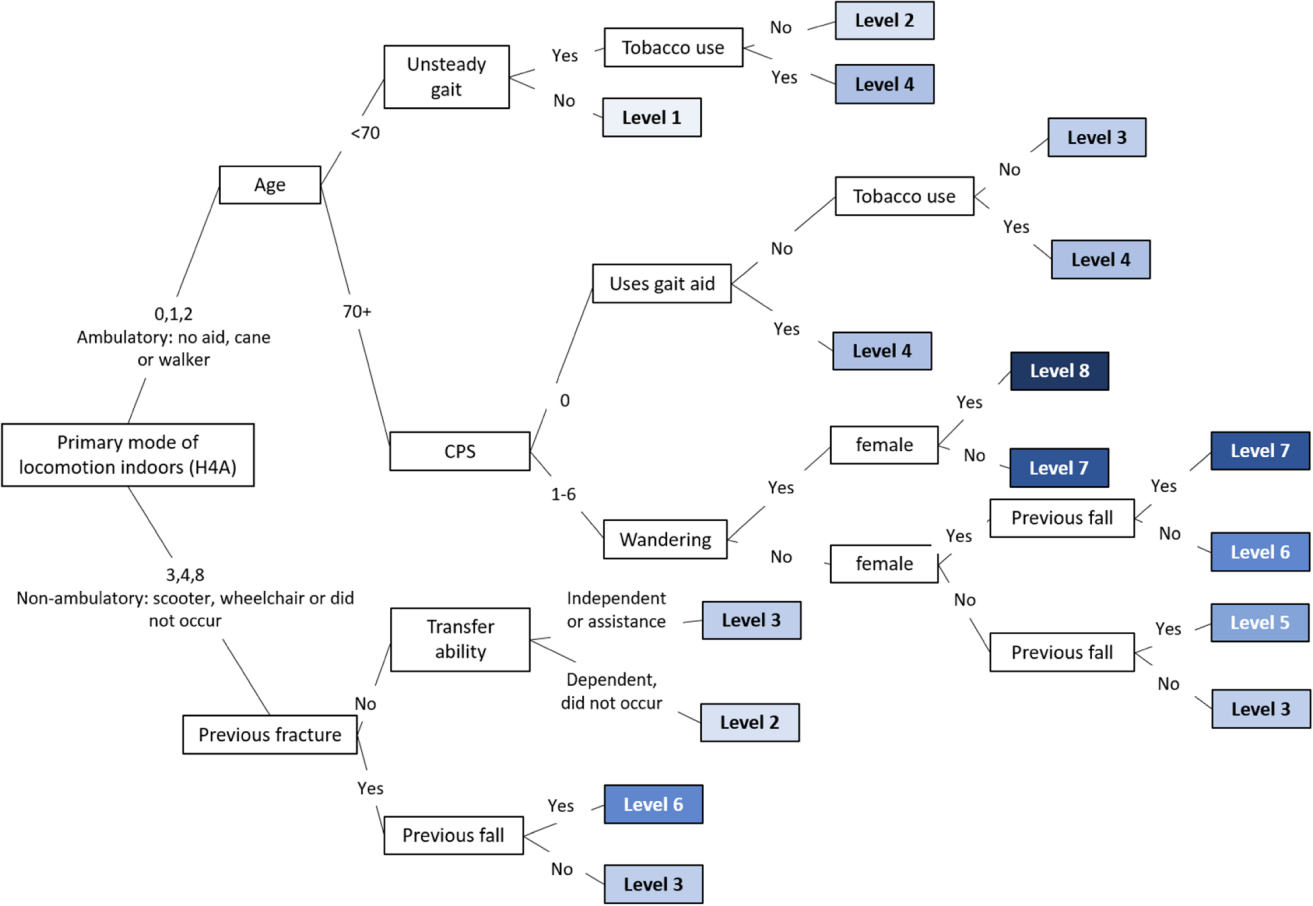
Fracture Risk Scale – Home Care. CPS=Cognitive Performance Scale

**Fig. 3:**
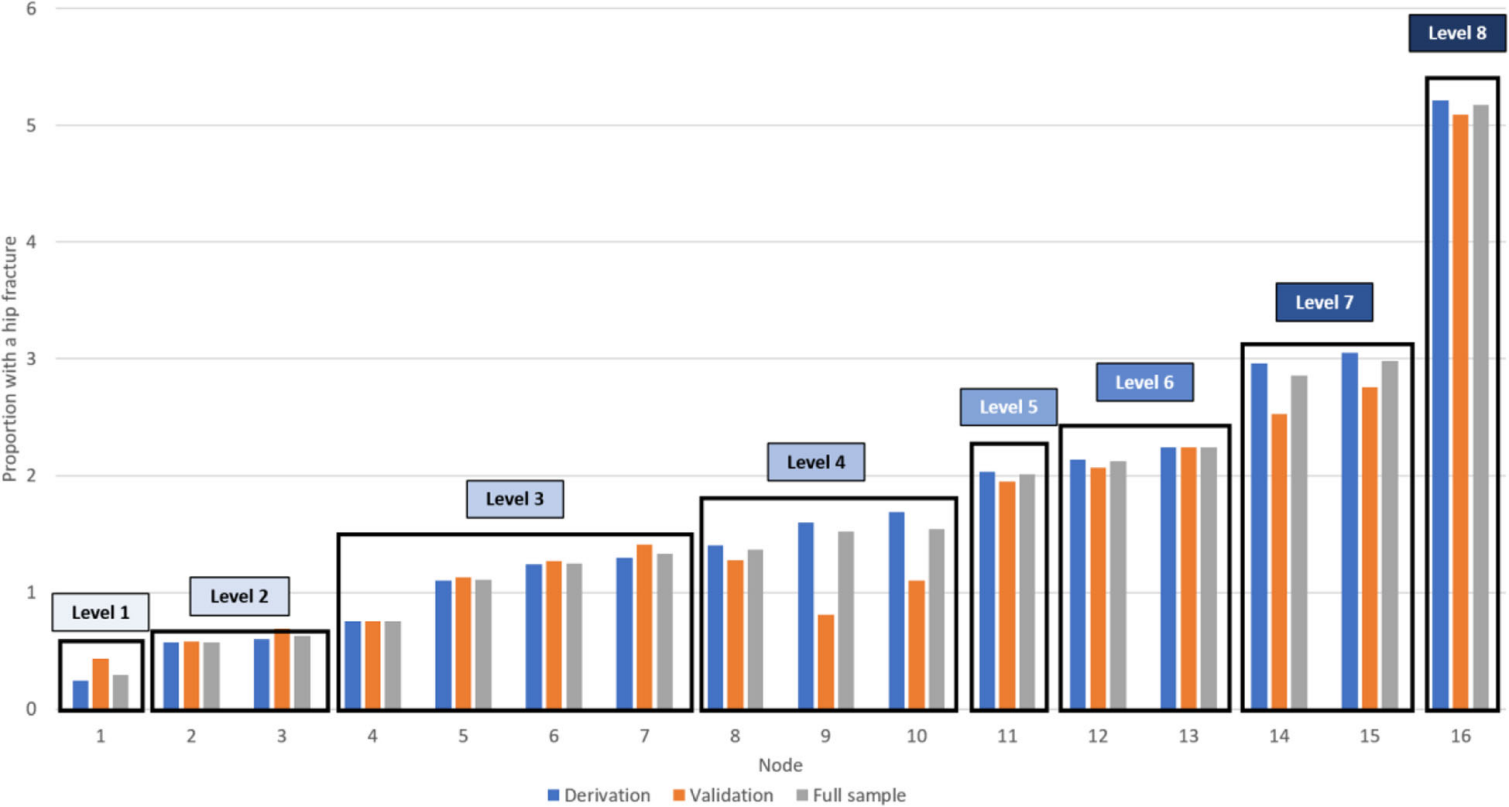
Incident hip fracture rates classified by individual decision nodes and the eight risk levels for the derivation, validation, and full sample datasets

**Fig. 4:**
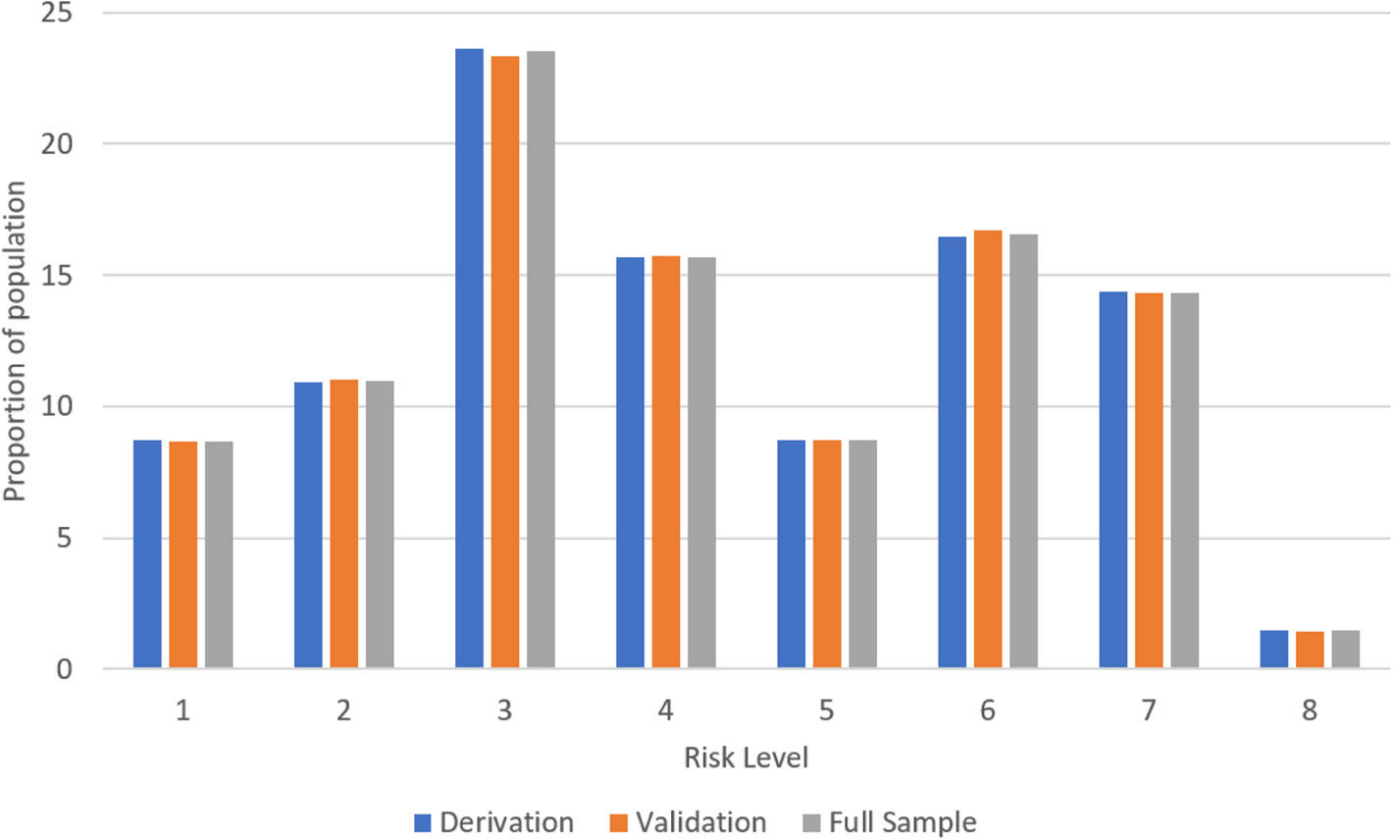
Distribution of home care recipients by hip fracture risk level for the derivation, validation, and full sample datasets

The final decision tree model had 16 leaves (Fig. 2) that were collapsed into the eight risk levels of the Fracture Risk Scale – Home Care (FRS-HC) (Fig. 3). The absolute proportion of home care recipients in each risk level who experienced a 1-year incident hip fracture ranged from 0.3 to 5.2% (Fig. 3). The odds of experiencing a fracture in each risk level as compared to the first level demonstrate a clear stepped progression, with the highest risk level having a 18.8-fold increase (95% confidence interval, 14.6 to 24.3) (Table 3). Figure 4 demonstrates that risk level 3 had the highest (23.6%) while risk level 6 had the lowest (1.5%) proportion of the population. Whether the individual was ambulatory or not demonstrated the highest discriminatory power in model, followed by age and experiencing a previous fracture in the past 180 days. Other variables included were unsteady gait, cognitive impairment, transfer ability, previous falls in the past 180 days, tobacco use, wandering, locomotion ability, and sex (Fig. 2).

**Table 3:** Odds of one-year incident hip fracture for the eight risk levels for the derivation, validation, and full sample

Hip fracture risk level categories	Derivation sample	Validation sample	Full sample
	OR (95% CI)	OR (95% CI)	OR (95% CI)
Level 2 vs 1	2.4 (1.8 to 3.3)	1.4 (0.9 to 2.2)	2.0 (1.6 to 2.6)
Level 3 vs 1	4.7 (3.5 to 6.2)	2.7 (1.9 to 4.0)	3.9 (3.1 to 5.0)
Level 4 vs 1	5.7 (4.2 to 7.6)	2.8 (1.9 to 4.1)	4.6 (3.6 to 5.8)
Level 5 vs 1	8.6 (6.4 to 11.5)	4.6 (3.1 to 6.8)	7.1 (5.6 to 8.9)
Level 6 vs 1	9.1 (6.8 to 12.1)	4.9 (3.4 to 7.2)	7.5 (6.0 to 9.5)
Level 7 vs 1	13.0 (9.8 to 17.3)	6.5 (4.4 to 9.4)	10.5 (8.4 to 13.2)
Level 8 vs 1	22.7 (16.6 to 31.2)	12.4 (7.9 to 19.2)	18.8 (14.6 to 24.3)

*OR* odds ratio, *CI* confidence interval

### Discrimination and predictive accuracy

Overall, the FRS-HC demonstrated good consistency between datasets. The discriminative properties of the FRS-HC were similar between the full sample (c-statistic = 0.658), derivation (c-statistic = 0.662), and validation (c-statistic = 0.645) datasets. Further, the absolute fracture rate for individual risk levels (Fig. 3) and the odds ratios between risk levels (Table 3) of the scale were similar between the derivation, validation, and combined datasets.

### Mortality rates

After a hip fracture, 9.5% (*N* = 479) of home care recipients died in the emergency department or as an inpatient after a mean length of stay of 20.2 days (standard deviation 38.6).

## Discussion

Home care recipients have a high risk of imminent hip fracture in the next year, which can result in pain, immobility, institutionalization, and death. We developed a Fracture Risk Scale for home care (FRS-HC) which predicts one-year incident hip fracture. Our results demonstrate that the FRS-HC can both discriminate and predict home care recipients at risk for hip fracture over a one-year time period. The FRS-HC score is calculated using routinely collected home care assessment data, includes clinically relevant information for medically complex home care recipients (e.g., falls, cognitive impairment, co-morbidities) and does not require additional information to be collected (e.g., bone mineral density) improving its clinical applicability and usefulness.

The FRS-HC predicts hip fracture within the next year, an important target for the vulnerable home care population. Indeed, fracture prevention strategies should be targeted to those at imminent risk for fracture including frail, older adults with a history of previous fractures and falls like the home care population [21]. Several currently available fracture risk assessment tools can determine imminent fracture risk. For example, QFracture [18] can calculate risk for any year between one and ten, while the Garvan fracture risk assessment tool [22] calculates risk for five and ten years. FRAX currently only calculates 10-year fracture risk, though it includes consideration for the competing risk of death as age increases [23]. As such, 10-year fracture risk as calculated by FRAX decreases with age because of the increasing likelihood of death. However, as age increases risk for fracture in the next year may be underestimated which could decrease the urgency for prevention strategies to be implemented [24]. The benefit of the FRS-HC is that it calculates fracture risk in the next year with data from routinely collected home care assessments thereby limiting additional documentation for an often overburdened sector.

The c-statistic of the FRS-HC is slightly lower than that of previously reported fracture risk assessment tools in other populations [25, 26]. However, caution must be taken when comparing c-statistics across studies. The c-statistic will vary depending on the characteristics of the cohort (e.g., age range) and length of follow-up [27, 28]. Further, the c-statistic does not increase with the addition of risk factors with strong predictive values but low prevalence which may be clinically relevant and aid in determining intervention thresholds [29]. Though it may be tempting to classify all home care recipients at high risk and implement widespread fracture prevention measures, this approach is not realistic in the home care sector. Prevention resources such as staff to support exercise and nutritional counselling are often limited and expensive in the home care sector. Further, home care recipients at low risk for fracture may receive minimal benefit from preventive strategies while unnecessarily consuming resources. Indeed, prevention is more cost-effective in high risk groups [30]. Targeting home care recipients who are truly at high risk will help with management of scarce resources for a sector with growing demands as the aging population increases. Though the c-statistic of the FRS-HC may be lower than previously developed fracture risk assessment algorithms we cannot compare across studies and regardless it will assist in allocating scare resource judiciously.

Our next step will be to develop a clinical assessment protocol (CAP) associated with the FRS-HC to link treatment and further investigation recommendations with the identified fracture risk. The CAP will classify home care recipients as low or high risk based on their FRS-HC score, and will provide evidence-based recommendations associated with their risk level. The FRS-HC identifies eight risk levels, with approximately 45% of the home care population in the lowest three risk categories [1–3] and fewer residents in the highest risk category (level 8). The lower distribution of home care recipients into higher risk categories is important as a large proportion of individuals identified as high risk can quickly overwhelm an overburdened sector, contribute to alarm fatigue, and cause many false positive identifications. Evidence-based care planning recommendations for the Fracture Risk CAP will be based on Canadian and international fracture prevention guidelines, and could include vitamin D and calcium supplementation, exercise, and pharmacological therapies [31]. The CAP will assist clinicians in identifying home care recipients at high fracture risk and recommended interventions to decrease that risk. Ultimately, the FRS-HC and associated CAP will reduce the risk of hip fracture, and healthcare costs, and improve quality of life. Given the substantial relationship between falls and fractures [32], integration of the current Falls CAP [33] and recently developed 1stFall algorithm [34], which predicts falls for home care recipients who have not previously fallen, will need to be explored. For example, it would be of value to examine the overlap between the rates that the FRS-HC and 1stFall scales each identify people at high risk, and between recommendations in the current Falls CAP and fracture prevention recommendations.

Our study has several limitations and strengths. We were limited to the inclusion of independent variables available in the RAI-HC, and we may not have captured all relevant fracture risk factors (e.g., body mass index). Further, we were only able to capture clinical vertebral fractures which likely represents 30% of all vertebral fractures during the one-year follow-up. Thus, our estimate for incidence of vertebral fractures is likely underestimated. We did not consider bone active medications in the analysis. We chose to develop the FRS-HC using routinely collected data to facilitate ease of implementation in the home care setting, and data on medications is not immediately available to the clinicians who would use the tool. Also, similar analyses done in a large cohort of community dwelling men revealed that exclusion of individuals on bisphosphonates did not alter the findings [35]. A strength of our study is the large number of data from home care recipients that was available for developing the scale. We also included a comprehensive list of independent variables in our analyses, confirmed by an expert panel. We used linked hospital data to confirm incident hip fractures. Decision tree analysis allowed us to develop a scale with an empirically sound, visual representation of the contributing factors to hip fractures for home care recipients. Further, since decision tree analysis has no parametric assumptions, it clusters risk factors, it has a better ability to account for outliers compared with regression analysis, and it often has higher utility in identifying high risk individuals [18, 19]. The FRS-HC uses items available in the RAI-HC and in the newer version of the assessment (interRAI HC). The RAI-HC and interRAI HC are used across several Canadian provinces and internationally, improving the usability and impact of our scale. Finally, the FRS-HC uses existing items from the RAI-HC, will automatically generate a fracture risk score based on routinely collected information, and will thereby decrease work duplication required to complete non-integrated tools such as FRAX.

## Conclusions

The FRS-HC predicts hip fracture over a one-year time period, demonstrates good discriminative and predictive properties, and can be used to support care planning by identifying home care recipients at high fracture risk. Future work should compare the FRS-HC to other fracture risk assessment tools, examine the relationship between the FRS-HC and falls scales, and develop a fracture risk clinical assessment protocol to link treatment recommendations with identified fracture risk.

## Data Availability

The data analyzed in this study are not publicly available due to privacy and confidentiality restrictions pertaining to person-level health information in Canada. However, the data set creation plan and underlying analytic code are available from the corresponding author on reasonable request.

## Abbreviations

RAI-HC: Resident Assessment Instrument – Home Care
FRS-HC: Fracture Risk Scale – Home Care
CAP: Clinical Assessment Protocol
NACRS: National Ambulatory Care Reporting System
DAD: Discharge Abstract Database
